# Use of a fractional dose of inactivated polio vaccine (fIPV) to increase IPV coverage among children under 5 years of age in Somalia

**DOI:** 10.1186/s44263-024-00044-7

**Published:** 2024-03-06

**Authors:** Khaliif Nouh, Abdirizak Haga, Kyandindi Sumaili, Muhammad Farid, Mohamed Alin, Mukhtar Shube, Abdirizak Abshir, Mohamed Hiirad, Muhyadeen Ahmed, Ahmed Bile

**Affiliations:** 1 United Nations Children’s Fund (UNICEF), Garowe, Somalia; 2 United Nations Children’s Fund (UNICEF), Mogadishu, Somalia; 3 World Health Organization (WHO), Mogadishu, Somalia; 4 Federal Ministry of Health (FMoH), Mogadishu, Somalia; 5 State Ministry of Health (SMoH), Puntland State, Garoowe, Somalia; 6 Somali Institute for Development Research and Analysis (SIDRA), London, UK

**Keywords:** Vaccination, Fractional inactivated polio vaccine, Campaign, Somalia

## Abstract

**Background:**

Global efforts reduced incidence of polio cases from 350,000 in 1988 to 22 cases in 2022 globally. There have been no wild poliovirus (WPV) cases seen in Somalia since August 2014. However, in 2017, there was a surge in the number of cases of circulating vaccine-derived poliovirus type 2 (cVDPV2), even with different intervention responses using monovalent oral polio vaccine type 2 (mOPV2). This study aimed to assess the use of fractional inactivated polio vaccine (fIPV), a smaller dose of the polio vaccine, equal to 1/5 of a standard dose, as an innovative polio vaccination delivery model, and identify the main opportunities for and challenges to the use of fIPV in the future for vaccinations.

**Methods:**

The study used two designs: a quasi-experimental design used to pilot fIPV in five districts and a cross-sectional study using both quantitative and qualitative approaches to collect primary data. A simple random sampling method was used to select 2 out of the 5 pilot districts for household surveys to study 768 participants. Key informant interviews and focus-group discussions were used to collect data from key frontline health workers and health/immunization officials involved in the campaigns. Secondary data from the pilot campaigns were analysed, such as administrative pilot data, lot quality assurance sampling (LQAS) and post-campaign communication assessments.

**Results:**

A total of 131,789 children aged 4–59 months were included for the pilot. Among these, 126,659 (96.1%) and 126,063 (95.6%) children were vaccinated in rounds 1 and 2, respectively. Out of the 768 households assessed, 99.9% had their children vaccinated. Nearly half of the few children who were not vaccinated were reported to be due to the parent of the child not being at home (48%). Ninety-seven percent of the qualitative study interviewees were satisfied with fIPV injection and recommended its use for routine immunization.

**Conclusions:**

The study findings are promising in the use of fIPV in mass campaigns to realize better coverage and global polio eradication. fIPV will potentially be used by policymakers in the design of polio eradication campaigns that integrate the fIPV vaccine into routine or supplementary immunization.

**Supplementary Information:**

The online version contains supplementary material available at 10.1186/s44263-024-00044-7.

## Background

Poliomyelitis is a highly infectious, acute communicable disease caused by a human enterovirus of the Picornaviridae family. Poliovirus is composed of a single-stranded, positive-sense RNA genome and a protein capsid and is transmitted from one person to another by oral contact with secretions or faecal material from an infected person. The virus is classified into wild poliovirus (WPV) and vaccine-derived poliovirus (VDPV). There are three serotypes of wild poliovirus that are antigenically distinct: type 1, type 2 and type 3. Most poliovirus infections cause asymptomatic viral replication that is limited to the alimentary tract. Some patients experience fever, fatigue, headache and sore throat, but paralytic poliomyelitis occurs in less than 1% of poliovirus infections, causing a condition called acute flaccid paralysis (AFP) and, in severe cases, resulting in death [1].

Polio eradication initiatives are among the key global public health priorities to deliver on the promise made at the 41st World Health Assembly (World Health Assembly, Geneva, 1988) to reduce and eliminate poliomyelitis from the world by the year 2000 [2]. Although the goal of a polio-free world by the year 2000 had not been achieved, global initiatives reduced the incidence of polio cases globally by 99% from an estimated more than 350,000 cases reported from 125 endemic countries in 1988 to 22 cases from two endemic countries in 2022 [3]. To ensure that the impact of polio is limited, some of the key strategies of polio eradication are to strengthen routine immunizations as well as to conduct large-scale National Immunization Days (NIDs). Political commitment, adequate resources, and capacities as well as effective planning and delivery of polio immunization services have been shown to be crucial contributors to the success of polio eradication initiatives [4, 5].

One of the major achievements of the global initiatives is the elimination of type 2 wild poliovirus, as no case of this type has been seen since 1999. Trivalent oral polio vaccine (tOPV) has been used to provide protection against all three types, but in April 2016, the type 2 component of the oral poliovirus vaccine (OPV) was withdrawn from routine immunization because of the risk of vaccine-derived poliovirus and vaccine-associated paralytic poliomyelitis, and the trivalent oral polio vaccine (tOPV) was replaced with a bivalent oral polio vaccine (bOPV) form [6]. Before the withdrawal of the tOPV, the Strategic Advisory Group of Experts on Immunization (SAGE) recommended that all countries should introduce at least one dose of inactivated polio vaccine (IPV) into their routine immunization schedule [7].

Very high priority is given to polio eradication activities and with a joint collaboration between Federal Ministry of Health (FMOH), State Health Ministries (SMOH), World Health Organization (WHO), United Nations Children’s Fund (UNICEF) and other partners. NID activities started in 1997, and many achievements were made in efforts to interrupt WPV transmission and strengthen disease surveillance. There have been no WPV cases seen in Somalia since August 2014.

Somalia fully switched from tOPV to bOPV in April 2016, and since then, OPV containing weakened strains of live poliovirus has been used in the country. However, one of the major challenges recorded after the switch was the emergence of cases of circulating vaccine-derived poliovirus (cVDPV) in different regions of Somalia. Vaccine-derived poliovirus is a well-documented strain of poliovirus mutated from the strain originally contained in OPV, which continues to affect different areas of the world. Cases of cVDPV are confirmed after the detection of VDPV that are genetically linked (> 0.6% nucleotide sequence divergence) in at least two different sources and at least 2 months apart, showing evidence of transmission in the community [8, 9]. In Somalia, there was one case of cVDPV reported in 2021, and two other cases were confirmed in 2022, as well as a number of confirmed environmental cases in both 2021 and 2022. Despite different interventions using mostly mOPV2 as an outbreak response, the cases have not yet been controlled, which is likely due to low coverage of routine immunizations in the country. According to WHO/UNICEF routine immunization estimates, 42% of children in Somalia received PENTA 3 (the 3rd dose of pentavalent vaccine containing diphtheria, pertussis, tetanus, hepatitis B and *Haemophilus influenzae*) in 2021 [10].

A fractional dose of inactivated polio vaccine (fIPV) contains the three types of polio vaccine, thus preventing all polioviruses (type I, type II and type III). fIPV is a smaller dose of inactivated polio vaccine, equal to 1/5 of a standard dose, and does not result in VDPV. Increasing polio immunization coverage rates is among the strategies to minimize the risk of wild poliovirus and cVDPV transmission. Since cVDPV cases are seen in various settings, particularly in countries where there are gaps in polio coverage, fIPV could be an option to be used alone or with OPV vaccines to increase coverage, stem cVDPV and realize the global polio eradication goals to protect children from this preventable disease. The findings of studies on the use of fIPV elsewhere show that it also has a significant role in outbreak response [11], and that there is no evidence of increased adverse events following immunization (AEFIs) with the use of the fractional dose [12]. Considering the importance of fIPV as an innovative delivery model, this study focussed on assessing the process and outcome of piloted campaigns and further understanding the acceptability of this vaccine among frontline healthcare workers and the community in five districts of Somalia: Berbera, Garowe, Dhusamareeb, Abdulaziz (in Mogadishu) and Dolow. In 2021, FMOH/SMOH, UNICEF, and WHO conducted two rounds of polio immunization campaigns using fIPV as a pilot delivery model in these five districts.

The efficacy of fractional doses of IPV has been studied since 1990 [11–26]. In recent years, evidence has grown to demonstrate that two fractional doses administered via the intradermal (ID) route offer higher immunogenicity than one full intramuscular (IM) dose of IPV [14–16, 27, 28]. A study published in 2018 showed that a single dose of fractional dose inactivated poliovirus vaccine (fIPV) boosted mucosal immunity to a similar degree as a full dose of IPV in children previously immunized with OPV [16]. Other studies concluded that two fractional doses of IPV administered by intradermal injection produced an even stronger immune response than a single full intramuscular IPV dose [12, 14, 15, 28–30].

The risk of strains such as cVDPV and imported WPV remains high and threatens children in countries with low rates of immunization, such as Somalia [31]. The fIPV intervention was conducted to increase immunization coverage against polio and reduce the risk of cVDPV from OPV. The campaign was successfully implemented in all five target districts of Berbera, Garowe, Dhusamareeb, Abdulaziz (in Mogadishu) and Dolow with very good coverage result. During September and October of 2022, we further studied and analyzed the fIPV pilot results and complemented with primary information from different sources using both quantitative and qualitative techniques to identify the main opportunities of this new vaccine since the previous results were encouraging, understand the status of community acceptance and possible challenges, and assess the potential use of fIPV as an innovative polio vaccine delivery model in supplementary and routine polio immunization to control and eliminate recurrent cVDPV cases seen in recent years.

## Methods

### Study settings

Berbera district, located in North West region, is the principal seaport in Somaliland and has a total population of 164,315. The city is located on the southern side of the Gulf of Aden.

Garowe is located in Nugal region and is the third largest city in Puntland State of Somalia. Geographically situated at the state’s centre, it is the seat of the Puntland government and has a total population of 205,735.

Dolow district is located in the northern part of the Gedo region of Jubbaland State of Somalia along the Jubba River. The town borders the Somali region in Ethiopia. Dolow has a total population of 93,275 and has recently hosted many IDPs.

Dhusamareeb, a district in Galguduud region, is located at the centre of Galmudug State of Somalia and is the capital city of the state, with a population of 139,980.

Abdi Aziz is a district among the 17 districts in the Banaadir region of Somalia (Mogadishu city). It lies on the Indian Ocean coast. Abdul-Aziz district has a total population of 55,640.

### Study design

The study used mixed methods, including quasi-experimental/interventional and cross-sectional methods [32, 33]. A quasi-experimental study was used to pilot fIPV vaccine, using the PharmaJet needle-free injector manufactured by PharmaJet company, based in Colorado, USA, in the five districts of Somalia. A cross-sectional design using both quantitative and qualitative approaches was used to determine the characteristics of the pilot population, assess the pilot outcome and obtain deeper insight into community experiences and perspectives about this model in relation to the administration and uptake of the polio vaccine at the pilot sites. Method and data source triangulation were employed, including capturing and analysing primary and secondary administrative data, lot quality assurance (LQAS) sampling, post-communication assessments of the fIPV piloting campaign, surveys with 768 households (HHs) in two selected districts (Garowe and Dolow) and 10 focus-group discussions (FGDs) and 12 key informant interviews (KIIs) with health workers and parents in three districts (Berbera, Garowe and Dolow).

### Study participants

The target population for the quantitative study was the parents/heads of households, while the KIIs and FGDs were conducted with key health/Expanded Program on Immunization (EPI) officials at the national, state, regional or district levels who were involved in the fIPV pilot campaigns which will restrict possible confounders related to fIPV knowledge and experience and with community members to further understand their perceptions of the fIPV system. The selection of the KII participants was based on their participation in recent fIPV campaigns and experiences of EPI in Somalia.

### Sampling

The selection of the two districts was performed purposively based on geographical representation. Garowe was selected as a sample to represent the northern districts, whereas Dolow is located in the southern part of Somalia. A two-stage sampling method was used for each of the two districts with the quantitative survey, where a sampling calculator was used for the first stage with a confidence interval of 95%, and random sampling was used in the second stage to select and assess 385 households in each district. For the qualitative study, nonprobability sampling was used to target health officials/health workers who participated in the campaigns and parents with < 5-year-old children whose children were immunized in the campaigns.

### Precampaign monitoring

Microplanning is the process of organizing the details of the operational plan, including resources and timeframe. Microplanning exercises were conducted for all five districts. The local routine immunization data together with official population statistics of the five districts have been studied to identify and estimate the target population, the required vaccine supplies, the workforce and logistics. Operational maps, vaccine supply management and the overall campaign implementation, monitoring and evaluation processes were all developed in the microplanning. The key personnel required for the campaign, such as vaccinators, social mobilizers, district field assistants (DFAs), supervisors and recorders, were trained. DFAs validated 100% of team-level micro plans. Using both a self-assessment desk and field validation checklist, each area team visited and verified the outreach and fixed sites, ensuring that the owner of the outreach site gave permission for the use of the property as an immunization site. The area teams assessed the capacity, accessibility and visibility of the sites and the availability of a referral hospital within the district. The micro plans contained contingencies such as buffer sites in case there was a need to replace the designated sites. Moreover, regional and country teams desk validated the micro plans.

### fIPV implementation in Somalia

Two fIPV immunization campaigns were conducted across five districts within five high-risk regions (one district per region) in Somalia, which were selected using risk modeling between September 2021 and December 2021. The target population for the campaign in the five districts was estimated to be 131,789 children aged 4–59 months. In contrast to OPV campaigns, in which house-to-house visits constitute the primary strategy for vaccination activities, the fIPV campaign was conducted at fixed sites, such as maternal and child health (MCH) centres, and through the deployment of teams to designated vaccination stations. Different social mobilization activities were undertaken by SOMNET, a social mobilization network for immunization in Somalia, with the support of the UNICEF social and behaviour change communications (SBCC) team before and during the campaigns. These activities included district social mapping, refusal monitoring and conversion, district communication plans (DCPs), nomadic movement tracking, printing and distribution of key information education and communication (IEC) messages and community orientations using sound trucks and house-to-house mobilizations.

The intervention consisted of two rounds of fIPV given at an 8-week interval, and a total of 131,789 children aged 4–59 months among the five selected districts were included. The vaccination activities took place at all designated locations during the 5-day campaign period. A team comprising two skilled persons (vaccinators), one OPV member, one team assistant, one recorder and one social mobilizer staffed each vaccination site. Vaccinators administered a 0.1-mL dose of fIPV intradermally.

The trainers of trainees (TOT) training for the campaigns was conducted in Mogadishu on 30 Aug 2021 with 20 participants and in Garowe on 14 September 2021 with 13 participants. This was followed by cascade training of 1334 health workers in 5 states of Somalia on the use of PharmaJet injectors, a device to deliver fractional IPV vaccines safely and effectively.

The district health teams, campaign supervisors and trained health workers collaborated to develop micro plans for fIPV’s supplementary immunization activities (SIAs) ahead of the campaigns. These micro plans included details of the specific number of children within the target age group for each location as well as management of vaccine and cold chain supplies. Social mobilizers were recruited to promote awareness of the campaigns in the districts where they were scheduled to take place. In addition, awareness of the campaign was promoted using posters and banners, radio and television, public announcements and the engagement of religious and community leaders.

During the 5-day period of the campaign, vaccination activities were carried out at appropriate sites from 8 a.m. to 4 p.m. These vaccination sites were located in public health facilities, and a few selected private health facilities with functioning EPI centres that offered routine EPI services and other antigens to eligible children during the campaign period. Temporary fixed sites were also used during the campaign. Vaccinators administered a 0.1-mL dose of fIPV (one-fifth of a full intramuscular dose of IPV, drawn from a vial containing 10 full intramuscular doses) intradermally in the upper left arm of each child, and assistants marked the left fifth finger of each vaccinated child with an indelible marker and entered records in a tally sheet.

### Data collection and analysis

Both primary and secondary data were used in this study. Household (HH) survey data, focus-group discussions (FGDs) and key informant interviews (KIIs) were collected and analyzed along with secondary data received from the fIPV piloting campaign, which included administrative data, LQAS, post-campaign independent monitoring and post-campaign communication assessments.

### Primary data

#### Quantitative approach

A structured survey questionnaire was developed to collect quantitative information from household heads/caregivers on their views on the new fIPV needle-free injector ([34, 35], Additional file 1). Two surveys were conducted with 768 caretakers in two districts (Garowe & Dolow) of the five piloted districts. Simple random sampling was used to ensure equal chance of participant selection, and the survey data was immediately collected within 1 month after the fIPV vaccine administration to avoid recall bias.

#### Qualitative approach

Key frontline health workers who had a good understanding and experience of the administration of fIPV and health care professionals who were involved in the implementation of the fIPV pilot campaigns as well as the parents who had children vaccinated in the campaigns were selected for the key informant interviews and focus-group discussions to explore their understanding, experiences, advantages, challenges and preferences in the fIPV campaigns [36, 37]. Twelve key informant interviews were conducted with key health workers, including MoH officials and members of partner organizations (Additional file 2).

There were four focus-group discussions with 32 vaccinators and social mobilizers who were among the teams that implemented the campaign and directly interacted with the parents and had good knowledge and experience in immunizations and could provide their perspectives on the administration of the vaccine, levels of community acceptance and uptake of the immunization ([38], Additional file 3). A total of 48 parents who had their children vaccinated with fIPV participated in six FGDs that were conducted alongside the quantitative surveys. Those parents also had experience with IPV vaccination.

### Secondary information

- fIPV campaign administrative data: Data from the WHO of both rounds of the fIPV pilot campaign were analysed to assess the coverage by each district and whether there were any variations between the two rounds of the campaign in any district and why.
- Lots quality assurance sampling (LQAS): LQAS was conducted after the campaign by independent groups with the support of the WHO. Sixty (60) households were assessed in each district to determine the proportion of children vaccinated and not vaccinated.
- Post-communication assessments: A study was also conducted after the campaign with 250 caregivers by the University of Bosaso (in Garowe) and Alpha University (in Hargeisa) with UNICEF support to understand the levels of awareness among the community before the campaign and to assess the effectiveness of each of the different communication methods used for the campaign to help plan and improve the information, education and communication for upcoming campaigns.
- Previous fIPV studies and experiences: Studies and experiences on fIPV from other countries were reviewed to identify research findings elsewhere in the world and compare them with the outcomes of the fIPV pilot campaigns in Somalia. The recommendations from different experts, including GPEI and WHO SAGE, were also reviewed.

### Data analysis

The primary survey data were collected in the open-source app Kobo Toolbox [39] and then downloaded into MS Excel [40]. The data were further cleaned and analysed using MS Excel and Statistical Package for Social Sciences (SPSS) version 21 [41]. Similarly, SPSS was used to analyse the secondary campaign data. Summary statistics such as the frequency and proportions of the variables of interest, including the fIPV coverage by administrative districts, reasons for missed children and source of campaign information, were generated and are presented in tables. The qualitative data were audio-recorded, transcribed in the Somali language and translated into English. The transcripts were coded, and thematic content was analysed separately [42]. The qualitative data were triangulated to develop a comprehensive understanding of fIPV implementation and campaign coverage and generate more robust results.

## Results

### fIPV vaccination strategies

Based on administrative data, a total of 131,789 children aged 4–59 months were included in two rounds (R1 and R2) of the fIPV vaccination campaign. A total of 126,659 and 126,063 children were reached in R1 and R2, respectively, showing coverage of 96% in each round. Variations were observed by district. Garowe, which reported a higher uptake of 111% for R1, recorded a lower coverage of 106% (5% decrease) in R2. This decrease was attributed to seasonal population movement, as many families who migrated from Bosaso city during the summer season returned to R2. In contrast, the districts of Berbera, Abdul-Aziz and Dhusamareb, which had a coverage less than 90% in R1, have seen an increase in R2. Berbera had a lower coverage of 88% in R1, as a large number of households migrated from Berbera due to hot summer weather but slightly increased in R2 (2% increase). The Dhusamareb district showed an improvement from 88% in R1 to 92% (4% increase) in R2. This correlates with the study findings that the interest in fIPV systems among the community increased in R2 even when the population movement patterns were accounted for [43] (see Table 1 on the proportion of children vaccinated in round 2 and Table 2 on the distribution of children by district in rounds 1 & 2).

**Table 1:** fIPV piloting: proportion of vaccinated children in R2 by district

**Target districts**	**Vaccinated 4–11 m 2nd dose total**	**Vaccinated 12–59 m 2nd dose total**	**Total vaccinated** (4**–**59 months) **2nd round**	**Percent of total vaccinated 2nd round**
Abdul-Aziz	1511	8040	9551	86%
Dusamareb	3709	22,047	25,756	92%
Dolow	4672	12,812	17,484	94%
Garowe	4944	38,705	43,649	106%
Berbera	7450	22,173	29,623	90%
Total	**22,286**	**103,777**	**126,063**	**96%**

**Table 2:** Distribution of vaccinated children by district and age groups — 1st and 2nd round of the fIPV campaign

District	Target children (4–59 months)	Round 1 vaccinated children		Round t2wo vaccinated children	
		**#**	**%**	**#**	**%**
Abdul-Aziz	11,128	9501	85%	9551	86%
Dusamareeb	27,996	24,680	88%	25,756	92%
Dolow	18,655	17,699	95%	17,484	94%
Garowe	41,147	45,743	111%	43,649	106%
Berbers	32,863	29,036	88%	29,623	90%
Total/coverage	131,789	126,659	96%	126,063	96%

Based on post communication assessment in Berbera, after R1, 98.7% of the target children were reached. Of the 1.3% of children who missed vaccination, nearly half of them missed due to the parent of the child not being at home (48%), vaccinators not coming to the house (37.9%) and parent/caregiver refusing (2.6%). This evidence indicates that the majority of children missed the vaccination for reasons other than refusal [44] (see Table 3 of reasons for missed children in Berbera district during round 1).

**Table 3:** Reasons for missed children in Berbera during round 1

Reasons for missed children	%
Household not visited by vaccinator	37.9%
Parent or child not at home	48.0%
Parent/caregiver refused	2.6%
Other reasons	11.5%

### fIPV preference

After the completion of fractional IPV campaigns in Garowe, a household survey on post-campaign communication was conducted. A total of 250 caretakers were interviewed about their views on fractional IPV and whether they would prefer it for their children. All 250 HHs (100%) recommended the use of fIPV in the future. Various reasons were recorded for the participant’s preference for fIPV systems. Over half (60.8%) of the 250 caregivers were motivated to use fIPV because there was no discomfort or pain experienced during injection, as with other types of vaccines. No struggle by children during administration constituted 26.8%, no worries since there was no injection (7.2%) and quick administration by health workers (5.2%) were the other reasons for preference for fIPV [45] (see Table 4 on why parents recommend fIPV use).

**Table 4:** Why parents recommend fIPV use

Why parents recommend fIPV use	#	%
Child did not struggle during the vaccination	67	26.8%
Health workers administered the vaccine quicker	13	5.2%
My child did not cry as usually does when injected	152	60.8%
Not worried, since no injection was involved	18	7.2%

### Demographic characteristics

Quantitative household assessments were performed in two (Garowe, and Dollow) out of the five districts, with 768 households assessed: 385 in Dolow and 383 in Garowe. The majority of the interviewees, 748 (97.4%), were female, and only 20 (2.6%) were men. The high number of female participants reflects the role of women mostly as mothers take care of young children in the Somalia context. Two-thirds of the interviewees (76%) were in second or third age groups of 20–29 (39.2%) and 30–39 (36.8%).

The results showed a low level of literacy among respondents, as more than half of the respondents reported that they had never been to school (see Table 5 on demographic characteristics of participants).

**Table 5:** Demographic characteristics of participants

**Gender**	**Garowe**	**%**	**Dolow**	**%**	**Total**	**%**
Female	378	98.7	370	96.1	748	97.4
Male	5	1.3	15	3.9	20	2.6
**Total**	**383**	**100**	**385**	**100**	**768**	**100**
**Age**	**Garowe**	**%**	**Dolow**	**%**	**Total**	**%**
< 20 years	17	4.4	13	3.4	30	3.9
20–29 years	156	40.7	145	37.7	301	39.2
30–39 years	163	42.6	120	31.2	283	36.8
40–49 years	39	10.2	67	17.4	106	13.8
≥ 50 years	8	2.1	40	10.4	48	6.3
**Total**	**383**	**100**	**385**	**100**	**768**	**100**
**Education**	**Garowe**	**%**	**Dolow**	**%**	**Total**	**%**
Not at school	135	35.2	270	70.1	405	52.7
Primary	118	30.8	109	28.3	227	29.6
Secondary	77	20.1	5	1.3	82	10.7
University	53	13.8	1	0.3	54	7.0
**Total**	**383**	**100**	**385**	**100**	**768**	**100**

### Precampaign awareness

Based on the quantitative survey, 638 (83.1%) out of the total survey 768 households reported that they received precampaign information, and over half of them (52.2%) gained awareness through a single information source. Among this group, the key sources of information about the campaign were social mobilizers (52.2%) and vaccinators/health workers (15%). Mass media, such as radio and TV, accounted for < 5% of caregiver campaign awareness.

### Reasons that motivated parents to vaccinate their children using fIPV

The main reason that motivated more than half of the respondents (51.4%) was to prevent their children from getting poliovirus, whereas 23.3% heard that the new vaccine was better than the one previously used, 21.3% said that they wanted their children to stay healthy, and 3.6% of the interviewees received encouragement after they had seen that their relatives and friends were bringing the children for vaccination. The proportion of parents who brought their children for immunization because they heard about the new delivery method was much higher in Dollow (42.6%) than in Garowe (3.9%) (see Table 6 on reasons that motivated parents to vaccinate their children using fIPV in RI).

**Table 6:** Reasons that motivated for vaccination and use of the injector for routine immunizations

	**Dolow**		**Garowe**		**Total**	
	**#**	**%**	**#**	**%**	**#**	**%**
Reason that motivated parents to vaccinate their children using fIPV						
I heard it is a new vaccine and better than the one already in use	164	42.6	15	3.9	179	23.3
I want my child to stay healthy	49	12.7	116	30.3	165	21.5
My relatives and friends were also bringing their children for vaccination	5	1.3	23	6.0	28	3.6
To keep/prevent my child from getting poliovirus	167	43.4	228	59.5	395	51.4
Other	0	0	1	0.3	1	0.1
**Total**	**385**	100	**383**	100	**768**	100
**Use this vaccine injector**						
Yes	385	100.0	360	94.0	745	97
No	0	0.0	23	6.0	23	3.0
**Total**	**385**	100.0	**383**	100.0	**768**	100.0

A total of 745 (97%) out of the 768 interviewees thought that all injectable vaccines should be administered using this needle-free injector, and only 3% of them thought that this was not needed.

In the key informant interviews, participants described their experiences with the implementation of the fIPV pilot as a fast and child-friendly delivery strategy. One respondent said that “the overall performance of the campaign was good, though seasonal issues may have had an impact”. Another participant observed that “the training given to all supervisors, vaccinators, and social mobilizers, and different methods of advocacy and social mobilizations had played an important role in the success of the pilot”. The majority of the participants agreed that “it was a very innovative polio delivery approach and commented that it was safe and easily administrable while the children were happy and smiling”.

Healthcare workers suggested that “fIPV was unique and different from other polio campaigns, as there was no pain and no delays during the implementation”. They said that “the fIPV had the potential to reach many more children as it could be implemented faster than other immunization methods”. Another health worker noted that “acceptance among the community rose very high quite quickly once parents were assured that it was child friendly, effective and safe”. She continued, “people told me that they preferred fIPV over IPV because of the needle-free injector and the painless administration”. Another health worker related “the increased community acceptance in the second round to better information, and community experience with the new needle-less and painless injector”.

A Ministry of Health official said that “the preparations of this supplementary polio immunization campaign in this particular delivery strategy of fIPV (trainings, social mobilization and awareness raising) was mostly similar to the previous polio campaigns with other modes but the only different element in this campaign was the use of needleless injector and adjusted dosing regimen”.

The majority of the key informants recommended introducing fIPV as routine immunization and a replacement for the current IPV since the coverage of polio vaccination with IPV is still low. They consider “the introduction of fIPV as a great opportunity to increase uptake as, unlike IPV, its acceptance among the community was high”. Their other suggestions included the use of fIPV as at least an SIAs mode that could support efforts to control the incidence and frequency of the observed cVDPV cases in Somalia.

In the focus-group discussions, the majority of the health workers concurred that fIPV was much safer since it was not an injection. They described how children struggle when intramuscular injection is administered and the risk of injecting the wrong position of the child’s body or injecting yourself accidentally due to the child’s movements. They also expressed their satisfaction with the fIPV since the children did not cry or struggle as the vaccines were administered, and the parents were happy with this delivery model. The health workers also said that by using fIPV, it was possible to reach more children in a short period.

Most health workers indicated that, in the beginning, they had worried about the use of the needle-free injector (PharmaJet) and had misgivings about acceptability among the community, but after they were trained, they realized that it was very friendly and could be used to vaccinate more children in a short time with minimal refusals. One health worker recalled that the “method was also a surprise to many parents who, as they were told that the injector was for polio vaccine, asked why it was not oral”. Another health worker said that “some of the parents had concerns about the safety of the vaccine because it was said to be a “pilot”. Parent’s worry was relieved, with the accurate information and after they saw that the child was not crying and not struggling”.

The perception of the health workers on the PharmaJet injector completely changed after the training and practice in the pilot. They readily offered suggestions for the injector to be used for routine immunizations.

In the focus-group discussions with the parents, participants reported that they worried at the beginning about the needle-free injector because they had never seen it before but later realized that it was painless and safe. Many of them described that they had very pleasant experiences with the vaccination, and that this injector was far better than the injection. One parent said that “it was common for the child to feel some fear at the outset, but once the vaccine was administered, they did not cry and were all smiling, so I would prefer the device more”. Another parent reflected that “when I had first seen PharmaJet, it was a surprise to me, and I felt fear for my child, but later I saw that it was normal and did not cause pain or any other harm. So I welcome the use of the needle free injector in the future for children regarding polio vaccination”. All the parents said “they preferred the needle-free injector (fIPV) over the injection (IPV) and agreed that it was painless, and the children did not cry and were happy to have it used in the future”.

### Challenges of fIPV Use

A few challenges were experienced by mothers and caregivers in fIPV use and implementation. Although no challenges were identified in the Dolow district, in Garowe district, few participants believed that there was pain associated with this route that stimulated the crying of children. Out of the 768 participants, 23 people (3%) reported that they were not happy with this injector because it was painful or believed that no vaccine was given to the child or that the same bottle was used for several children. There were two major challenges that the subjects in this study mentioned: (i) the initial fear of the new technology and the fact that caregivers heard about the project being piloted, and (ii) social mobilization messages were not adequately tailored to promote new technology (needleless injector) and fIPV but rather focused on polio vaccination. When asked, the health authority said that they wanted to avoid further confusing the community, which had already shown fatigue in receiving several new vaccines regularly.

## Discussion

The ongoing global initiatives to achieve a polio-free world drive the search for and development of innovative technological solutions and novel delivery models for routine and supplementary polio immunization programmes. Although vaccination campaigns using injectable vaccines against other infectious diseases have been carried out globally, in Somalia, this was the first campaign to use fIPV. Overall, the study findings demonstrated that it was viable to plan and implement the fIPV campaign and achieve higher coverage in Somalia. These findings were comparable to a study in Pakistan, which reported high coverage of greater than 82% [20], and results from other studies, which indicated that over 97% of vaccinators and parents preferred the use of needleless injectors for polio vaccination [21, 22].

There was some initial hesitancy among some mothers regarding the use of the needleless injector because “the technique and vaccine itself were new to us”. However, it was later shown that the administration of the vaccine was much easier and painless than IPV injection. Regarding the performance of the campaign between the two rounds, the coverage in the first round (R1) was slightly lower than that in the second round (R2) in most of districts, which was mainly attributed to limited information and experience with the new vaccine delivery model. Health workers reported that this type of vaccine was new to the community, but the parents accepted the vaccine to be given to their children, although there was some fear among them at the beginning. In addition, the health workers themselves had similar fears of the gadget, the needleless injectors (PharmaJet), which were newly presented to them despite the training, exposure and practice before the commencement of the campaign. Hence, the health workers adopted the new technique later on and performed better during the second round of the campaign in November 2021. The increased uptake of the fIPV in the second round (R2) points towards an enhanced or deepened community acceptance of the delivery method in light of favourable experience with the needle-free administration method as well as better vaccine delivery techniques by health care workers using the needle-free injector. A similar experience was reported in the fIPV campaign conducted in Karachi, Pakistan, using the same needleless injectors from PharmaJet in 2019. Thus, it was reported that the performance was promising, and parents showed twice in favour of fIPV compared to IPV vaccination [22]. A respondent in this study said, “The new technique for the vaccine was very good and simple injection, and the health workers and the parents preferred this new technique”. However, the loading of the new gadget, the PharmaJet [30], was not easy, as another respondent pointed out that health workers struggled to do that at the beginning. Despite the initial concerns by parents and teething problems with the gadgets by health care workers, the evidence from this study suggests that the impressively high coverage post-campaign was mainly due to preference for the use of intradermal needle-free injectors in addition to other factors, including the low quantity of vaccine required, the speed and ease of administration by vaccinators and the willingness of parents to bring their children due to the painless administration of the vaccine.

While this study did not examine the cost-effectiveness of this method or the level of immunity produced, our findings in relation to high coverage and community acceptance are supported by research findings in clinical trials in Oman [13, 32], Cuba [46], Bangladesh [28, 30] and Sri Lanka [23] and mass campaigns in Pakistan [20, 22, 24] and China [25], which reported the impact of fIPV on large-scale coverage of 85.3% as well as the immunogenic potential of two doses of fIPV to be superior or on part with one dose of IPV. A study in Telangana, India, also gave credence to the results of these trial campaign results and went further to recommend that “lessons learned in the campaign can be applied in future outbreak responses using fIPV” [26]. Our results are further supported by GPEI [12] and are in line with the guidance of the WHO Strategic Advisory Group of Experts on Immunization (SAGE) in the WHO position paper on polio vaccines [47].

Regarding precampaign awareness, the majority of the interviewed households received information about the campaign from social mobilizers who performed house-to-house mobilizations before the campaign. Other sources of the information included sound trucks, mosque announcements and health teams [44, 45]. In the community acceptance and perception of the use of needless injectors and fIPV vaccination, it was noted that there was no difference between the pilot campaign approaches and the typical polio vaccination awareness-raising campaigns in the past, and the community did not notice any difference. It was also noted that health education activities were mainly confined to towns and were not well visible in rural communities. Some respondents suggested that “mothers were satisfied with the painless injectors regardless of the initial fear” and reported that the time of the campaign was convenient, and that administration was smooth and timely. One said, “The safety and effectiveness are the same with the IPV, it is only the new technique that we need the health workers to adopt”.

The study had some strengths and limitations. The main strength of the study is related to the large sample size and random sampling method that was used to select households as per standard World Health Organization (WHO) RCA survey methodology, thereby minimizing the occurrence of selection bias. In the cross-sectional component of the study, it was possible that some parents might not have shared actual reasons for refusing to vaccinate their children, which could create social desirability bias. In general, a lack of accurate population estimates and numerator and denominator information pose challenges for setting target populations and planning, monitoring and evaluating interventions in Somalia. Thus, there is always a risk of over- or underestimation in the administrative EPI data. Despite these limitations, this study is the first to explore fIPV implementation in Somalia, and its findings support the use of intradermal needle-free injectors.

The findings of this study present similar conclusions to those conducted before in different countries (Pakistan, India, Cuba, Bangladesh, Oman), which are all encouragingly in favour of the use of fIPV over IPV [12, 24–26, 28]. This delivery method could offer an opportunity for Somalia and similar settings where the coverage of routine immunizations is low, especially with IPV, and in interventions to overcome cVDPV cases reported every year.

The participants of the study were very favourable to the prospect of using fIPV in routine immunization because “mothers preferred this type of vaccine since it is considered to be safe, painless, and easy to administer”. Furthermore, they recommended that social mobilization should be conducted well before the campaign and should specifically promote the additional benefits that intradermal fIPV administered through the new technology brings into the polio vaccination programme. Finally, they emphasized that the new needle-free injector should be used for the delivery of other routine vaccinations that would attract mothers and boost coverage.

Although the initial price of the injector is high, it is a one-off cost. Two doses of fIPV will likely be more cost effective than one full dose of intramuscular IPV [12, 27] because of the cost of reduced doses. Where a 5-ml IPV vial delivers 10 doses of IPV, it can provide 25 doses when used in fIPV. Moreover, some supplies, such as syringes and safety boxes, are not needed for fIPV, which will likely reduce the cost.

It is important to note that strong government leadership at the national and state levels, well-coordinated technical and operational support from UNICEF, WHO and partners, clearly defined standard operating procedures for the intervention, and the well-established experience of implementing OPV campaigns in the past was critical elements to the success of the fIPV campaign in Somalia.

However, debates about the viability of the fIPV delivery method are related to not only the preference for needleless administration, which results in high coverage, but also the efficacy of dose-adjusted regimens (smaller doses), cost-effectiveness and logistics of different delivery strategies. According to GPEI 2017 [12], a two-dose fIPV schedule has been strongly recommended to countries by the WHO Strategic Advisory Group of Experts on Immunization (SAGE) and in the WHO position paper on polio vaccines [47]. SAGE used evidence from studies to support their recommendations that fIPV is safe, effective and immunogenic; it can be given alone or at the same time as any other vaccine; it can be used in all types of polio immunization activities: routine immunization, SIAs and outbreak response; and finally, in children also receiving oral polio vaccine (OPV), two doses of fIPV given at 6 and 14 weeks will help to “boost” their mucosal immunity against polioviruses [12, 29, 47, 48].

The results support the use of fIPV to increase IPV coverage and efforts to control recurrent cVDPV2 cases in Somalia. The main challenge could be the availability of needleless PharmaJet injectors; however, if those devices are secured, the adoption of fIPV delivery methods will likely present a good opportunity to reach more children with vital immunization services in Somalia. Therefore, the study recommends possible options, such as using needle-free injectors in routine immunization as there is an opportunity to include two doses of fIPV in RI schedules instead of IPV. This will likely increase the coverage since the acceptance of fIPV is much higher than that of IPV. Another possibility is to use it as a mode of supplementary campaigns to ensure reaching a large number of target children, which will also increase immunity and will likely support efforts to limit the re-emergence and spread of cVDPV2 cases. And finally, if any of the above two options are not possible, it could be used at least in high-risk districts, locations with recent cVDPV2 or districts with a high number of zero cases.

## Conclusions

The study concludes that a fractional dose of IPV by using a needle-free injector for the administration of polio vaccine was highly approved by the community living in the five districts. The majority (97%) of caregivers proposed using this injector instead of injection. The use of intradermal needle-free injectors was supported because the injector was easy to use by vaccinators, the administration of the vaccine was painless as the children did not cry and no delays were experienced during the implementation. The findings of the study present crucial information to fill the information gap concerning improving polio immunization uptake to achieve polio-free Somalia and support the exploration and use of innovative delivery methods in routine immunization as well as in mass campaigns to produce a level of herd immunity capable of curtailing the proportion of paralytic cases of poliovirus because it was possible to achieve high vaccination coverage with a fractional dose of IPV. This strategy could be replicated by EPI/polio immunization planning and policy makers at the Federal and State Ministries of Health to implement the recommendations to introduce fIPV in routine polio immunization and outbreak interventions in Somalia. The success of the fIPV pilot campaigns in Somalia also strengthens the argument for further research and clinical trials on the immunogenicity and cost-effectiveness of the fIPV delivery model.

## Supplementary Information

**Additional file 1.**

**Additional file 2.**

**Additional file 3.**

**Additional file 4.**

## Data Availability

The datasets used and/or analyzed during the current study are contained within this article. All the raw data including qualitative and quantitative or clarification are available upon reasonable request to protect participants’ privacy and confidentiality, by contacting the corresponding author by emailing to nuux71@yahoo.com.

## Abbreviations

AEFI: Adverse effect following immunization
bOPV: Bivalent oral polio vaccine
cVDPV: Circulating vaccine-driven poliovirus
DCPs: District communication plans
DFAs: District field assistants
EPI: Expended programme on immunizations
FGDs: Focus-group discussions
fIPV: Fractional dose of inactivated polio vaccine
FMOH: Federal Ministry of Health
GPEI: Global Polio Eradication Initiatives
HHs: Households
ID: Intradermal
IDPs: Internally displaced people
IEC: Information education and communication
IM: Intermuscular
KIIs: Key informant interviews
LQAS: Lots quality assurance sampling
MCHs: Maternal and child health
mOPV2: Monovalent oral polio vaccine (type 2)
NIAGs: National Islamic Advisory Group
NIDs: National Immunization Days
OPV: Oral polio vaccine
R1: Round 1 (fIPV campaign)
R2: Round 2 (fIPV campaign)
RCA: Root cause analysis
RI: Routine immunization
SAGE: Strategic Advisory Group of Experts
SBCC: Social and behaviour change communications
SPSS: Statistical Package for Social Science
SIAs: Supplementary immunization activities
SMOH: State Ministry of Health
SOMNET: Social mobilization networks
tOPV: Trivalent oral polio vaccine
TOT: Trainers of trainees
UNICEF: United Nations Children’s Fund
VDPV: Vaccine-driven poliovirus
WHO: World Health Organization
