# Supplementary material for: Use of a fractional dose of inactivated polio vaccine (fIPV) to increase IPV coverage among children under 5 years of age in Somalia
Source: BMC Glob Public Health. 2024 Mar 6;2:16. doi: 10.1186/s44263-024-00044-7 (PMC11622934; doi:10.1186/s44263-024-00044-7)
Supplement: Supplementary file 1 — Additional file 1. [file 44263_2024_44_MOESM1_ESM.docx]

**Fractional dose of inactivated polio vaccine (fIPV) piloting in Somalia to increase IPV coverage among children under five years of age**

## Household Questionnaire

Care-givers or parents

**Section A: Demagogic status of Caregiver/parent:**

| Age: |  | | |
| --- | --- | --- | --- |
| Sex: | 🞎 Male | | 🞎 Female |
| Education Level: | |  | |

1. How did you hear about the fIPV campaign? (Maximum of 2 sources)

🞎 From a health workers/social mobilizer

🞎 From sound truck announcement

🞎 From radio/TV announcement

🞎 From Mosques announcement

🞎 From Peer Friends or Family member

🞎 Other (specify)…………….

1. What motivated you to bring your child for vaccination: (select only one)

🞎 To keep/prevent my child from getting Polio Virus.

🞎 I want my child to stay healthy

🞎 My relatives and friends were also bringing their children for vaccination

🞎 I heard it is a new vaccine and better than the one already in use

🞎 Other (specify)

1. What was your reaction when you realized that the vaccinators were going to use a device different from needle and syringe to vaccinate your child?

🞎 I was worried and asked them for explanation before allowing them

🞎 I was worried but asked no questions

🞎 I was worried somewhat but I trusted that the vaccinators knew how to do the right thing

🞎 I was not worried at all because I heard from other people about the new device which the health workers were using

1. How do you feel now that your child was vaccinated using the new device?

🞎 I feel happy, my child did not cry as he/she usually does when injected

🞎 I am happy as there was no struggle to administer the vaccine

🞎 I am quite surprised about the speed with which the vaccine was given

🞎 I am not happy because I don’t think any vaccine was given to my child

1. Do you think that (if possible) all injectable vaccines should be administered using this device?

🞎 Yes 🞎 No

1. If Yes, why?

🞎 It is painless and my child did not cry

🞎 Nurses administer the vaccination faster using this device

🞎 …………………………………..

🞎 Other (please specify)…………………………………

1. If No, why not?

🞎 Nurses administer the vaccination more slowly using this device

🞎 The same bottle was used for several children

🞎 I am not happy because I don’t think any vaccine was given to my child

🞎 Other (please specify)…………………………………

Thank YOU
