# Supplementary material for: Use of a fractional dose of inactivated polio vaccine (fIPV) to increase IPV coverage among children under 5 years of age in Somalia
Source: BMC Glob Public Health. 2024 Mar 6;2:16. doi: 10.1186/s44263-024-00044-7 (PMC11622934; doi:10.1186/s44263-024-00044-7)
Supplement: Supplementary file 2 — Additional file 2. [file 44263_2024_44_MOESM2_ESM.docx]

**Fractional dose of inactivated polio vaccine (fIPV) piloting in Somalia to increase IPV coverage among children under five years of age**

**Key Informants Interview Topic Guide**

| Name: |  | Age |  |
| --- | --- | --- | --- |
| Occupation: |  | District |  |
| Role: |  | Date: |  |

1. Did you participate the fIPV piloting campaigns in last year (yes/no)?

If yes, according to your observations how was the overall performance of fIPV campaign in this district.____________________________________________________________________________________________________________________________________________________________________________________________________________________________________

Coild you please explain your answer to the overall performance of fIPV?

________________________________________________________________________________________________________________________________________________________________________________________________________________

1. What was your impression about fIPV as an innovate polio immunization delivery model? ________________________________________________________________________________________________________________________________________________________________________________________________________________________________________________________________________________________________________________________
2. How was the fIPV pilot conducted in terms of information/communication, advocacy, and education? What kind of information and education were given to communities prior the campaign? Was it different in any way from the other polio campaigns? ________________________________________________________________________________________________________________________________________________________________________________________________________________________________________________________________________________________________________________________
3. Was there any different or unique features and factors of the fIPV pilot compared to the other polio immunization campaigns?

________________________________________________________________________________________________________________________________________________________________________________________________________________________________________________________________________________________________________________________

1. How were the perception/acceptances of the community about fIPV? __________________________________________________________________________________________________________________________________________________________________________________________________________________________________________
2. Were there any variations between the two campaigns when it comes to community acceptance? and if yes explain the differences? What do you think was the reason of difference?

____________________________________________________________________________________________________________________________________________________________________________________________________________________________________________________________________________________________________________________________________________________________________________________________________

1. What are the differences between fIPV and IPV regarding community acceptance and feasibility of utilizations? _______________________________________________________________________________________________________________________________________________________________________________________________________________________________________________________________________________________________________________________
2. How satisfied were you with the fIPV immunization?

Very satisfied

Satisfied

Very dissatisfied

Dissatisfied

I do not know

If you were very satisfied or satisfied, why? __________________________________________________________________________________________________________________________________________________________________________________________________________________________________________

If you were very dissatisfied or dissatisfied, why? __________________________________________________________________________________________________________________________________________________________________________________________________________________________________________

­­­­­­­­­

1. Please choose one of the following statements

There was sufficient information about the campaign Yes  No

The campaign was conducted in a convenient time / date Yes  No

The vaccine was easy to administer Yes  No

The vaccine was easy to receive Yes  No

It did not take long time to administer it Yes  No

It did not take long time to receive it Yes  No

1. What were the main challenges seen during the campaign?

________________________________________________________________________________________________________________________________________________________________________________________________________________________________________________________________________________________________________________________

1. Do you think fIPV can be used as routine immunization? Why? Why not? ________________________________________________________________________________________________________________________________________________________________________________________________________________________________________________________________________________________________________________________
2. What are the benefits and opportunities of using fIPV as routine immunization?

__________________________________________________________________________________________________________________________________________________________________________________________________________________________________________

1. What were the main barriers (in your opinion) that could hinder children getting vaccinated against polio?
   - - - - Probe: social, cultural, or gender-related barriers and those relating to the availability, accessibility and delivery of immunization services.
2. Would you recommend other methods of utilizing fIPV other than routine immunization, if yes, what are those methods? __________________________________________________________________________________________________________________________________________________________________________________________________________________________________________
3. What is your recommendation on fIPV? __________________________________________________________________________________________________________________________________________________________________________________________________________________________________________
