# Supplementary material for: Use of a fractional dose of inactivated polio vaccine (fIPV) to increase IPV coverage among children under 5 years of age in Somalia
Source: BMC Glob Public Health. 2024 Mar 6;2:16. doi: 10.1186/s44263-024-00044-7 (PMC11622934; doi:10.1186/s44263-024-00044-7)
Supplement: Supplementary file 3 — Additional file 3. [file 44263_2024_44_MOESM3_ESM.docx]

## **Fractional dose of inactivated polio vaccine (fIPV) piloting in Somalia to increase IPV coverage among children under five years of age**

## Focus Group Discussion Topic Guide

**Invite health workers who have used the device and those who were part of the fIPV campaign**

1. Can you please tell us about your role in the fIPV polio vaccination campaign and the MoH/health facility / organisation you work with and in what capacity (your role)? (e.g., MoH, UN, International and Local NGO?
2. Before the training, what did you feel about the device? (training)
3. What was your experience like during the administration of the vaccine with the new device (experience on the use of the device)?
   1. Use probing questions to ask more about administering the vaccine such as ease of use, usefulness, time, etc.
4. What was the reaction from the parents/caregivers and how did the children feel? Was it painful or painless? (parents and children’s reaction?)
5. What were different in the fIPV campaign from other previous polio campaigns?
   1. Use probing questions to gain their perspective on how the campaign was conducted (the process, information, mobilization, etc.) and features/factors/components that could have made fIPV successful/unsuccessful
6. What were the main barriers (in your opinion) that could hinder children getting vaccinated against polio?
   1. Probe: social, cultural, or gender-related barriers and those relating to the availability, accessibility and delivery of immunization services.
7. What do you think were the advantages and challenges of using the device and this mode of vaccine delivery?
8. Which one would you prefer between the AD syringes and needle-free injectors? Use probing questions to allow the participants to give reasons? (AEFI, vaccine wastage, volume sharps, etc)

## **Fractional dose of inactivated polio vaccine (fIPV) piloting in Somalia to increase IPV coverage among children under five years of age**

## Focus Group Discussion Topic Guide

**Invite parents / caregivers of children vaccinated with the new device and those who were part of the campaign**

1. Can you please tell us about your role in the fIPV polio vaccination campaign (caregiver, mother, father, grandparent, community member)
2. What was your experience and the experience of your children like during the administration of the vaccine with the new device (experience on the use of the device)
3. How did the children feel about the administration of the vaccine with fIPV? Did the children feel pain? Did the children cry, feel discomfort or struggle? (parents’ observation about the children’s reactions)
4. What were different in the fIPV campaign from other previous polio campaigns?
   1. Use probing questions to gain their perspective on how the campaign was conducted (the process, information, mobilization, etc) and features/factors/components that could have made fIPV successful/unsuccessful
5. What were the main barriers (in your opinion) that could hinder children getting vaccinated against polio?
   1. Probe: social, cultural, or gender-related barriers and those relating to the availability, accessibility and delivery of immunization services.
6. Which one would you prefer between the AD syringes and needle-free injectors? Use probing questions to allow the participants to give reasons.
