# Supplementary material for: Use of a fractional dose of inactivated polio vaccine (fIPV) to increase IPV coverage among children under 5 years of age in Somalia
Source: BMC Glob Public Health. 2024 Mar 6;2:16. doi: 10.1186/s44263-024-00044-7 (PMC11622934; doi:10.1186/s44263-024-00044-7)
Supplement: Supplementary file 4 — Additional file 4. [file 44263_2024_44_MOESM4_ESM.docx]

Informed Consent Form (English)

**Research Title:** **Fractional dose of inactivated polio vaccine (fIPV) piloting in Somalia to increase IPV coverage among children under five years of age**

**Name of PI/Researcher responsible for project: Khaliif Nouh**

**Participant Identification Number:**

| **Statement** | **Please circle** |
| --- | --- |
| I agree to take part in the above-named study and understand that I can refuse to answer questions and that I can withdraw from the study at any time without having to give a reason. I have read (or been read to) and understood the study information or it has been read to me. I have been able to ask questions about the study and my questions have been answered to my satisfaction. | YES / NO |
| I agree to have my contributions recorded by the researchers. I understand that the recording will be stored safely and will be destroyed 3 years after the end of the project. | YES / NO |
| I understand that the information I provide will be used in research publications and to produce recommendations on polio eradication initiatives in Somalia and globally and that the information will be anonymised. | YES / NO |
| For focus group discussion participants: I agree to keep membership and discussions of the focus group confidential | YES / NO |

Participant name: ________________________________________________________

Signature:  ________________________________  Date: ______________________

I attest that I have explained the study information accurately in Somali to, and was understood to the best of my knowledge by, the participant and that he/she has freely given their consent to participate* in the presence of the below named impartial witness (where applicable).

Person taking consent name: __________________________________________________

Signature: ________________________________  Date: _______________________

For information please contact: Khaliif Nouh

Please retain the Original (x1) in site file and offer a Copy (x1) to the participant

* please offer the study information sheet to the participant
